# A novel technique to solve nonlinear higher-index Hessenberg differential–algebraic equations by Adomian decomposition method

**DOI:** 10.1186/s40064-016-2208-3

**Published:** 2016-05-11

**Authors:** Brahim Benhammouda

**Affiliations:** Abu Dhabi Men’s College, Higher Colleges of Technology, P.O. Box 25035, Abu Dhabi, United Arab Emirates

**Keywords:** Differential–algebraic equations, Adomian decomposition method, Analytical methods, 34A09, 65L80, 37Mxx

## Abstract

Since 1980, the Adomian decomposition method (ADM) has been extensively used as a simple powerful tool that applies directly to solve different kinds of nonlinear equations including functional, differential, integro-differential and algebraic equations. However, for differential–algebraic equations (DAEs) the ADM is applied only in four earlier works. There, the DAEs are first pre-processed by some transformations like index reductions before applying the ADM. The drawback of such transformations is that they can involve complex algorithms, can be computationally expensive and may lead to non-physical solutions. The purpose of this paper is to propose a novel technique that applies the ADM directly to solve a class of nonlinear higher-index Hessenberg DAEs systems efficiently. The main advantage of this technique is that; firstly it avoids complex transformations like index reductions and leads to a simple general algorithm. Secondly, it reduces the computational work by solving only linear algebraic systems with a constant coefficient matrix at each iteration, except for the first iteration where the algebraic system is nonlinear (if the DAE is nonlinear with respect to the algebraic variable). To demonstrate the effectiveness of the proposed technique, we apply it to a nonlinear index-three Hessenberg DAEs system with nonlinear algebraic constraints. This technique is straightforward and can be programmed in Maple or Mathematica to simulate real application problems.

## Background

Systems of differential–algebraic equations (DAEs) are often used to model many important problems in real applications. These equations arise, for instance, in electrical networks, optimal control, mechanical systems, incompressible fluids dynamics and chemical process simulations. DAEs are characterized by the so called index, which has several definitions (Günther and Wagner 2001; Kunkel and Mehrmann 1996; Martinson and Barton 2000). The most used index is the differentiation index, which is the minimum number of times that all or a part of the DAE must be differentiated with respect to time, in order to obtain an ordinary differential equation (Martinson and Barton 2000). In real applications, higher-index DAEs (differentiation index greater than one) arise naturally in many important application problems like constrained multibody systems (Benhammouda and Vazquez-Leal 2015; Simeon 1996; Simeon et al. 1994), vehicle system dynamics (Simeon et al. 1991), space shuttle simulation (Brenan 1983) and incompressible fluids dynamics. Unfortunately, higher-index DAEs are known to be difficult to solve. Therefore, they are usually transformed to ordinary differential systems (index-zero) or index-one DAEs before solving them. This transformation, called index-reduction, can be computationally very expensive and may also change the properties of the solution. Therefore, new techniques are required to solve higher-index DAEs efficiently.

The Adomian decomposition method (ADM) and its modifications (Adomian and Rach 1985; Adomian 1988; Almazmumy et al. 2012; Fatoorehchi et al. 2015; Hendi et al. 2012; Pue-on and Viryapong 2012; Ramana and Raghu Prasad 2014; Wazwaz 2001) are known to be efficient methods in solving a large variety of linear and nonlinear problems in science and engineering. Among these problems, we mention algebraic equations (Adomian and Rach 1985), ordinary differential equations (Almazmumy et al. 2012; Fatoorehchi et al. 2015; Hendi et al. 2012; Pue-on and Viryapong 2012; Ramana and Raghu Prasad 2014; Wazwaz 2001), partial differential equations (Adomian 1984) and integral equations (Bakodah 2012).

However, for the application of the ADM to DAEs, one finds only four pieces of work in the literature (Duan and Sun 2014; Çelika et al. 2006; Hosseini 2006a, b). In Duan and Sun (2014), an index-one DAE is transformed to a second order ordinary differential equation before applying the ADM to it. In Çelika et al. (2006), the ADM is applied to simple semi-explicit index-one DAEs, where the DAE is first transformed to a system of ordinary differential equations before applying the ADM. In Hosseini (2006a), the ADM is applied to linear higher-index Hessenberg DAEs after transforming them to index-one DAEs. In Hosseini (2006b), index-one and index-two DAEs with linear constraints are solved where these DAEs are pre-processed by a transformation that relies much on the special forms they have. Therefore, in all these previous works, the ADM is not applied directly to the DAEs but rather to the transformed equations. The drawback of such transformations is that they can involve complex algorithms, can be computationally expensive and may lead to non-physical solutions.

In this work, we present a new procedure for solving a class of nonlinear higher-index Hessenberg DAEs based on the ADM. The ADM is first applied directly to the DAE where the nonlinear terms are expanded using the Adomian polynomials (Duan 2010a, b, 2011, Rach 2008, 1984; Wazwaz 2000). Based on the index condition, a nonsingular algebraic recursion system is derived for the expansion components of the solution. Also, it is important to note that unlike previous works (Duan and Sun 2014; Çelika et al. 2006; Hosseini 2006a, b), our procedure does not make transformations to the DAEs before applying the ADM to them. To demonstrate the effectiveness of the proposed technique, we solve a nonlinear index-three Hessenberg DAEs system with nonlinear algebraic constraints. Further, our technique is based on a simple algorithm that can be programmed in Maple or Mathematica to simulate real application problems.

This paper is organized as follows: in “Review of the Adomian decomposition method” section, we review the ADM for solving ordinary differential equations. Next, in “The proposed method” section, we present our method for the solution of nonlinear higher-index Hessenberg DAEs systems. Then in “Application to a nonlinear index-3 DAEs system” section, we apply the developed technique to solve a nonlinear index-three Hessenberg DAEs system with nonlinear algebraic constraints. Finally, a discussion and a conclusion are given in “Discussion” and “Conclusion” sections, respectively.

## Review of the Adomian decomposition method

In this section, we give a brief review for the Adomian decomposition method (ADM) (Adomian and Rach 1985; Almazmumy et al. 2012; Fatoorehchi et al. 2015; Hendi et al. 2012; Pue-on and Viryapong 2012; Ramana and Raghu Prasad 2014; Wazwaz 2001) to solve ordinary differential equations. For this purpose, let us consider the following nonlinear differential equation

1 $$\begin{aligned} Lu+Ru+N(u)=f, \end{aligned}$$

where *L* is an easily invertible operator (usually taken as the highest-order derivative), *R* is an operator grouping the remaining lower-order derivatives, $N\left( u\right)$ is the nonlinear term and *f* is a given analytical function.

Solving Eq. (1) for *Lu* then applying the inverse operator $L^{-1}$ to both sides, we obtain

2 $$\begin{aligned} L^{-1}Lu=L^{-1}f-L^{-1}Ru-L^{-1}N\left( u\right) . \end{aligned}$$

If $Lu=\dot{u}=du/dt$ and the initial condition $u\left( t_{0}\right) =c_{0}$ is given, then $L^{-1}$ represents the integral from $t_{0}$ to *t* and $L^{-1}Lu=u-c_{0}.$ If $Lu=\ddot{u}=u^{(2)}=d^{2}u/dt^{2}$ and the initial conditions $u\left( t_{0}\right) =c_{0}$ and $\dot{u}\left( t_{0}\right) =c_{1}$ are given, then $L^{-1}$ is the double fold integral from $t_{0}$ to *t* and $L^{-1}Lu=u-c_{0}-c_{1}\left( t-t_{0}\right)$. In this case from (2), we have

3 $$\begin{aligned} u=g+L^{-1}f-L^{-1}Ru-L^{-1}N\left( u\right) , \end{aligned}$$

where $$g=c_{0}+c_{1}\left( t-t_{0}\right) .$$

To apply the ADM to Eq. (3), we first assume that the solution *u* of (1) to have the infinite series form

4 $$\begin{aligned} u=\sum _{n=0}^{\infty }u_{n}, \end{aligned}$$

where the unknown solution components $$u_{n},$$$$n=0,1,2,\ldots$$ are to be determined later by the method.

Second, the nonlinear term *N*(*u*) is expanded in an infinite series in terms of the Adomian polynomials $N_{n}$ (Duan 2010a, b, 2011; Rach 2008, 1984; Wazwaz 2000) as

5 $$\begin{aligned} N\left( u\right) =\sum _{n=0}^{\infty }N_{n}\left( u_{0},\ldots ,u_{n}\right) . \end{aligned}$$

Substituting (4) and (5) into (3) and choosing $u_{0}$ as

6 $$\begin{aligned} u_{0}=g+L^{-1}f, \end{aligned}$$

we obtain

7 $$\begin{aligned} \sum _{n=0}^{\infty }u_{n}=u_{0}-L^{-1}R\sum _{n=0}^{\infty }u_{n}-L^{-1}\sum _{n=0}^{\infty }N_{n}. \end{aligned}$$

Comparing the general terms on the left hand side and the right hand side of (7), we derive the following recursion scheme for the ADM

8 $$\begin{aligned} u_{n}=-L^{-1}Ru_{n-1}-L^{-1}N_{n-1},\quad n\ge 1. \end{aligned}$$

Since $u_{0}$ is known from (6), recursion (8) can be used to generate as many solution components $u_{n}$ as one wants. Further, if series (4) converges then it gives the exact solution of (1) and an approximation of order *K* to solution can be obtained from

9 $$\begin{aligned} u(t)=\sum _{n=0}^{K-1}u_{n}. \end{aligned}$$

To compute the Adomian polynomials $N_{n}$, $n=0,1,\ldots$ associated with the nonlinearity $N\left( u\right) ,$ one can use the following definition for all forms of nonlinearity

10 $$\begin{aligned} N_{n}\left( u_{0},\ldots ,u_{n}\right) =\frac{1}{n!}\frac{d^{n}}{d\lambda ^{n}}\left[ N\left( \sum _{i=0}^{\infty }\lambda ^{i}u_{i}\right) \right] _{\lambda =0},\quad n\ge 0. \end{aligned}$$

Using this formula, we obtain the following first few Adomian polynomials

11 $$\begin{aligned} N_{0}\left( u_{0}\right) &= N\left( u_{0}\right) , \\ N_{1}\left( u_{0},u_{1}\right)&= u_{1}N^{\prime }\left( u_{0}\right) , \\ N_{2}\left( u_{0},u_{1},u_{2}\right)&= u_{2}N^{\prime }\left( u_{0}\right) + \dfrac{u_{1}^{2}}{2!}N^{\prime \prime }\left( u_{0}\right) , \\ N_{3}\left( u_{0},u_{1},u_{2},u_{3}\right)&= u_{3}N^{\prime }\left( u_{0}\right) +u_{1}u_{2}N^{\prime \prime }\left( u_{0}\right) +\dfrac{u_{1}^{3}}{3!}N^{\prime \prime \prime }\left( u_{0}\right) , \\ N_{4}\left( u_{0},u_{1},u_{2},u_{3},u_{4}\right)&= u_{4}N^{\prime }\left( u_{0}\right) +\left( \dfrac{u_{2}^{2}}{2!}+u_{1}u_{3}\right) N^{\prime \prime }\left( u_{0}\right) +\dfrac{u_{1}^{2}u_{2}}{2!}N^{\prime \prime \prime }\left( u_{0}\right) \\&+\dfrac{u_{1}^{4}}{4!}N^{(4)}\left( u_{0}\right) , \end{aligned}$$

where the dash $(^{\prime })$ represents the differentiation with respect to $u_{0}$.

In a similar manner, one can easily generate the remaining polynomials from ( 10) and expand (11). In the literature, there are several algorithms for computing the Adomian polynomials without the need for formula (10), but a more convenient algorithm for the *m*-variable case is recently proposed in Duan (2011)

12 $$\begin{aligned} N_{n}=\dfrac{1}{n}\sum _{k=1}^{m}\sum _{i=0}^{n-1}\left( i+1\right) v_{k,i+1} \frac{\partial N_{n-1-i}}{\partial v_{k,0}},\quad n\ge 1. \end{aligned}$$

## The proposed method

In this section, we present our method for solving a class of nonlinear higher-index Hessenberg differential–algebraic equations (DAEs). This technique is based on the Adomian decomposition method (ADM). To solve this class of DAEs, we first apply the ADM directly to it and expand the nonlinear terms using the Adomian polynomials. Then, an algebraic recursion system for the solution expansion components is derived. Taking account of the index of the DAE, this system is shown to be uniquely solvable for the solution expansion components. Also, it is important to note that unlike previous works (Çelika et al. 2006; Duan and Sun 2014, 2006a, b), our technique does not make transformations to DAEs before applying the ADM to them.

The class of nonlinear higher-index Hessenberg DAEs we consider here is

13 $$\begin{aligned} \left\{ \begin{array}{l} u^{\left( m\right) }\left( t\right) =M\left( u\left( t\right) ,\quad v\left( t\right) \right) , \\ 0=N\left( u\left( t\right) \right) ,\quad t\ge t_{0}, \end{array} \right. \end{aligned}$$

where $u^{\left( m\right) }\left( t\right)$ stands for $d^{m}u/dt^{m}$, $m\,\ge 1$ and $u\in \mathbb {R}^{n_{u}}$, $v\in \mathbb {R}^{n_{v}}$, $M: \mathbb {R}^{n_{u}}\times \mathbb {R}^{n_{v}}\longrightarrow \mathbb {R} ^{n_{u}}$, $N:\mathbb {R}^{n_{u}}\longrightarrow \mathbb {R}^{n_{v}}$.

This DAE is supplied with some consistent initial conditions

14 $$\begin{aligned} u^{\left( n\right) }\left( t_{0}\right) =c_{n}, \end{aligned}$$

where $c_{n}$, $n=0,\ldots ,m-1$ are given constants. Note here that no initial conditions are prescribed to the variable *v*. We also assume that DAE initial-value problem (13)–(14) has a unique analytical solution.

The vectors *u* and *v* are called the differential and the algebraic variables respectively. System (13) is index $(m+1)$ if the square matrix

15 $$\begin{aligned} \dfrac{\partial N}{\partial u}\cdot \dfrac{\partial M}{\partial v}\in \mathbb {R}^{n_{v}}\times \mathbb {R}^{n_{v}} \end{aligned}$$

is nonsingular for $t\ge t_{0}.$

Systems (13) arise frequently in many important applications like Navier–Stokes equations in incompressible fluids dynamics or Euler–Lagrange equations in constrained multibody systems. In what follows, we assume that system (13) is index $(m+1)$ that is $(m+1)$-index condition (15) holds.

To solve DAE initial-value problem (13)–(14) by the ADM, we apply the operator $L^{-1}$ (which is in the present case the *m*-fold integral from $t_{0}$ to *t*) to both sides of the first equation of (13) to get

16 $$\begin{aligned} u=\sum _{n=0}^{m-1}\left( c_{n}/n!\right) \left( t-t_{0}\right) ^{n}+L^{-1}M\left( u,v\right) . \end{aligned}$$

First, we assume the components *u* and *v* of the solution of (13) to have the infinite series form

17 $$\begin{aligned} u=\sum _{n=0}^{\infty }u_{n},\quad v=\sum _{n=0}^{\infty }v_{n}, \end{aligned}$$

where the unknowns $$u_{n}$$ and $$v_{n},$$$$n=0,1,2,\ldots$$ will be determined later by our method.

Second, we expand the nonlinear terms $M\left( u,v\right)$ and *N*(*u*) in infinite series using the Adomian polynomials $M_{n}$ and $N_{n}$ as

18 $$\begin{aligned} M\left( u,v\right) =\sum _{n=0}^{\infty }M_{n},\, \, N\left( u\right) =\sum _{n=0}^{\infty }N_{n}, \end{aligned}$$

where $$M_{n}:=M_{n}\left( u_{0},v_{0},\ldots ,u_{n},v_{n}\right)$$ and $$N_{n}:=N_{n}\left( u_{0},\ldots ,u_{n}\right) .$$

Substituting expansions (17) and (18) into equation (16), we get

19 $$\begin{aligned} \left\{ \begin{array}{l} \sum \nolimits _{n=0}^{\infty }u_{n}=\sum \nolimits _{n=0}^{m-1}\left( c_{n}/n!\right) \left( t-t_{0}\right) ^{n}+\sum \nolimits _{n=0}^{\infty }L^{-1}M_{n}, \\ 0=\sum \nolimits _{n=0}^{\infty }N_{n}. \end{array} \right. \end{aligned}$$

Choosing the first *m* terms $u_{n}$ as

20 $$\begin{aligned} u_{n}=\left( c_{n}/n!\right) \left( t-t_{0}\right) ^{n},\, \, n=0,\ldots ,m-1, \end{aligned}$$

then comparing the general term on the left hand side with that on the right hand side of (19), we derive the following recursion system for the unknowns $u_{n}$ and $v_{n-m}$

21 $$\begin{aligned} \left\{ \begin{array}{l} u_{n}=L^{-1}M_{n-m}, \\ 0=N_{n},\quad n\ge m. \end{array} \right. \end{aligned}$$

Using (12), the Adomian polynomials $M_{n}$ and $N_{n},\,n=0,1,2,\ldots$ can be written as

22 $$\begin{aligned} M_{n}=\left\{ \begin{array}{l} M\left( u_{0},v_{0}\right) ,\quad n=0, \\ \dfrac{1}{n}\sum \nolimits _{i=1}^{n-1}i\left( \dfrac{\partial M_{n-i}}{\partial u_{0}} \right) u_{i}+i\left( \dfrac{\partial M_{n-i}}{\partial v_{0}}\right) v_{i}+\left( \frac{\partial M_{0}}{\partial u_{0}}\right) u_{n}+\left( \frac{ \partial M_{0}}{\partial v_{0}}\right) v_{n},\quad n\ge 1, \end{array} \right. \end{aligned}$$

and

23 $$\begin{aligned} N_{n}=\left\{ \begin{array}{l} N\left( u_{0}\right) ,\quad n=0, \\ \dfrac{1}{n}\sum \nolimits _{i=1}^{n-1}i\left( \dfrac{\partial N_{n-i}}{\partial u_{0}} \right) u_{i}+\left( \frac{\partial N_{0}}{\partial u_{0}}\right) u_{n},\quad n\ge 1. \end{array} \right. \end{aligned}$$

One important property of the Adomian polynomials $M_{n}$ and $N_{n}$ we exploit here is their linearity with respect to $v_{n}$ and $u_{n},\,n\ge 1$ respectively.

For the value $n=m,$ system (21) can be written as

24 $$\begin{aligned} \left\{ \begin{array}{l} u_{m}-L^{-1}M\left( u_{0},v_{0}\right) =0, \\ -\left( \frac{\partial N_{0}}{\partial u_{0}}\right) u_{m}=\dfrac{1}{m} \sum \nolimits _{i=1}^{m-1}i\left( \dfrac{\partial N_{m-i}}{\partial u_{0}}\right) u_{i}. \end{array} \right. \end{aligned}$$

The unknowns in this algebraic system are $u_{m}$ and $v_{0}.$ System (24) is linear if the given function $M\left( u,v\right)$ is linear with respect to *v* and this system is nonlinear with respect to $v_{0}$ if $M\left( u,v\right)$ is nonlinear with respect to *v*. Since index condition (15) holds, the Jacobian matrix

25 $$\begin{aligned} \left( \begin{array}{cc} I &{} -\frac{\partial M_{0}}{\partial v_{0}} \\ -\frac{\partial N_{0}}{\partial u_{0}} &{} 0 \end{array} \right) \end{aligned}$$

of system (24) is nonsingular. Therefore, system (24) is uniquely solvable for the unknowns $u_{m}$ and $v_{0}$.

To solve system (24) for $u_{m}$ and $v_{0}$, we multiply its first equation by $-\frac{\partial N_{0}}{\partial u_{0}}$ and substitute $-\left( \frac{\partial N_{0}}{\partial u_{0}}\right) u_{m}$ by its expression from the second equation of (24). Then, we obtain the following equation for the unknown $v_{0}$

26 $$\begin{aligned} \left( \frac{\partial N_{0}}{\partial u_{0}}\right) L^{-1}M\left( u_{0},v_{0}\right) =-\dfrac{1}{m}\sum _{i=1}^{m-1}i\left( \dfrac{\partial N_{m-i}}{\partial u_{0}}\right) u_{i}. \end{aligned}$$

Since index condition (15) holds, Eq. (26) can be solved uniquely for $v_{0}$. Substituting this computed value of $v_{0}$ into the first equation of (24), we can determine $u_{m}.$

Now for the values $n\ge m+1,$ we use the first equation of (21) and the second equation of (22) to get

27 $$\begin{aligned} u_{n}-\left( \frac{\partial M_{0}}{\partial v_{0}}\right) w_{n-m}=P, \end{aligned}$$

where

28 $$\begin{aligned} w_{n-m}&= L^{-1}v_{n-m}, \\ P&= L^{-1}Q, \\ Q&= \dfrac{1}{n-m}\sum _{i=1}^{n-m-1}i\left( \dfrac{\partial M_{n-m-i}}{ \partial u_{0}}\right) u_{i}+i\left( \dfrac{\partial M_{n-m-i}}{\partial v_{0}}\right) v_{i}+\left( \frac{\partial M_{0}}{\partial u_{0}}\right) u_{n-m}. \end{aligned}$$

From the second equations of (21) and (23), we deduce that

29 $$\begin{aligned} -\left( \frac{\partial N_{0}}{\partial u_{0}}\right) u_{n}=R,\quad n\ge m+1, \end{aligned}$$

where

30 $$\begin{aligned} R=\dfrac{1}{n}\sum _{i=1}^{n-1}i\left( \dfrac{\partial N_{n-i}}{\partial u_{0} }\right) u_{i}. \end{aligned}$$

Set of Eqs. (27) and (29) is a linear algebraic system for the unknowns $u_{n}$ and $w_{n-m}.$ Since index condition (15) holds, the coefficient matrix (25) of this system is nonsingular. Therefore, system (27) and (29) is uniquely solvable for $u_{n}$ and $w_{n-m},$ for $n\ge m+1$.

To solve set of Eqs. (27) and (29), we multiply Eq. (27) by $-\frac{\partial N_{0}}{\partial u_{0}}$ and substitute $-\left( \frac{\partial N_{0}}{\partial u_{0}}\right) u_{n}$ by its expression from (29), to obtain the following algebraic system for the unknown $w_{n-m}$

31 $$\begin{aligned} \left( \frac{\partial N_{0}}{\partial u_{0}}\cdot \frac{\partial M_{0}}{\partial v_{0}}\right) w_{n-m}=-R+\left( \frac{\partial N_{0}}{\partial u_{0} }\right) P. \end{aligned}$$

Since index condition (15) holds, Eq. (31) can be solved uniquely for $w_{n-m}$, and we have

32 $$\begin{aligned} w_{n-m}=\left( \frac{\partial N_{0}}{\partial u_{0}}\cdot \frac{\partial M_{0}}{\partial v_{0}}\right) ^{-1}\left[ -R+\left( \frac{\partial N_{0}}{ \partial u_{0}}\right) P\right] . \end{aligned}$$

Then, substituting the expression of $w_{n-m}$ into (27), we determine the unknown $u_{n}$

33 $$\begin{aligned} u_{n}=\left( \frac{\partial M_{0}}{\partial v_{0}}\right) \left( \frac{\partial N_{0}}{\partial u_{0}}\cdot \frac{\partial M_{0}}{\partial v_{0}} \right) ^{-1}\left[ -R+\left( \frac{\partial N_{0}}{\partial u_{0}}\right) P \right] +P. \end{aligned}$$

Now differentiating Eq. (32) *m* times with respect to *t*, we determine the unknown $v_{n-m}$

34 $$\begin{aligned} v_{n-m}=\left( \frac{\partial N_{0}}{\partial u_{0}}\cdot \frac{\partial M_{0}}{\partial v_{0}}\right) ^{-1}\left[ -R^{\left( m\right) }+\left( \frac{ \partial N_{0}}{\partial u_{0}}\right) P^{\left( m\right) }\right] . \end{aligned}$$

Using (33) and (34) for $n\ge m$ with (20), we can determine all solution components $u_{n}$ and $v_{n}.$ An approximate solution for DAE initial-value problem (13)–(14) can be obtained using (9) as

35 $$\begin{aligned} u(t)=\displaystyle {{\sum _{n=0}^{K-1}}}u_{n},\, \, v(t)=\displaystyle {{ \sum _{n=0}^{K-m-1}}}v_{n}, \end{aligned}$$

where *K* is the order of approximation of *u*(*t*).

It is worth noting that Eq. (21) is linear with respect to $v_{n-m}$ for all values of $n\ge m$, except for the case $n=m$ where (21) is nonlinear with respect to $v_{0}$ if the given function *M*(*u*, *v*) is nonlinear with respect to *v*. This linearity property of Eq. (21) has a great positive impact on the simplicity of our method and its efficiency. Note here also that many important problems arising from applications like constrained mechanical systems and the semi-discrete form of Navier–Stokes equations lead to DAEs systems of the form of (13 ), where *M*(*u*, *v*) is linear with respect to *v*. For these problems, system ( 21) is linear for all values of $n\ge m$.

## Application to a nonlinear index-3 DAEs system

In this section, we illustrate and demonstrate the effectiveness of our technique to solve nonlinear higher-index Hessenberg DAEs systems, which are known to be difficult to solve even numerically. Following the procedure developed in the previous section, we first apply the ADM directly to the DAEs system and expand the nonlinear terms using the Adomian polynomials. Then, taking account of the index of the DAE, we derive a nonsingular algebraic recursion system for the expansion components of the solution. Finally, by solving this algebraic system we obtain the solution of the DAE.

As a test problem, we consider the following nonlinear index-three Hessenberg DAEs system which describes the constrained motion of a particle to a circular track Benhammouda (2015)

36 $$\begin{aligned} \ddot{u}_{1}&= 2u_{2}-2u_{2}^{3}-u_{1}v, \\ \ddot{u}_{2}&= 2u_{1}-2u_{1}^{3}-u_{2}v, \\ 0&= u_{1}^{2}+u_{2}^{2}-1,\quad t\ge 0. \end{aligned}$$

DAEs system (36) is supplied with the following (consistent) initial conditions

37 $$\begin{aligned} u_{1}\left( 0\right) =1,\quad \dot{u}_{1}\left( 0\right) =0,\quad u_{2}\left( 0\right) =0,\quad \dot{u}_{2}\left( 0\right) =1. \end{aligned}$$

Note that no initial condition $v\left( 0\right)$ is prescribed to the variable $v\left( t\right)$ because $v\left( 0\right)$ is pre-determined by the DAE and initial conditions (37). System (36) is index-three since three time differentiations of the algebraic equation (third equation) of (36) will lead to an ordinary differential equation for $v\left( t\right)$. As a consequence, this DAEs system is difficult to solve numerically. Note also that the third equation of (36) is nonlinear which make this DAEs system harder to solve.

To solve DAEs initial-value problem (36)–(37) by the procedure developed in the previous section, we let $Lu=\ddot{u}$ and have $L^{-1}u=\int _{0}^{t}\int _{0}^{t}udtdt.$

We assume that the solution components $$u_{1},$$$$u_{2},$$ and *v* of (36) to have the form

38 $$\begin{aligned} u_{1}=\sum _{n=0}^{\infty }u_{1,n},\quad u_{2}=\sum _{n=0}^{\infty }u_{2,n} ,\quad v=\sum _{n=0}^{\infty }v_{n}, \end{aligned}$$

where the unknowns $u_{1,n}$, $u_{2,n}$ and $v_{n}$, $n=0,1,2,\ldots$ will be determined later by our technique.

Applying $L^{-1}$ to both sides of the first two equations of (36), we obtain

39 $$\begin{aligned} u_{1}&= 1+L^{-1}\left( 2u_{2}-2u_{2}^{3}-u_{1}v\right) , \\ u_{2}&= t+L^{-1}\left( 2u_{1}-2u_{1}^{3}-u_{2}v\right) , \\ 0&= u_{1}^{2}+u_{2}^{2}-1,\quad t\ge 0. \end{aligned}$$

Substituting expressions (38) into (39), we get

40 $$\begin{aligned} \sum _{n=0}^{\infty }u_{1,n}&= 1+L^{-1}\sum _{n=0}^{\infty }\left( 2u_{2,n}-2A_{2,n}-B_{1,n}\right) , \\ \sum _{n=0}^{\infty }u_{2,n}&= t+L^{-1}\sum _{n=0}^{\infty }\left( 2u_{1,n}-2A_{1,n}-B_{2,n}\right) , \\ 0&= \sum _{n=0}^{\infty }C_{n}, \end{aligned}$$

where $A_{k,n},\,B_{k,n},\,k=1,2$ and $C_{n},\,n=0,1,2,\ldots$ are the Adomian polynomials associated to the nonlinear terms $u_{k}^{3},\,u_{k}v$ and $u_{1}^{2}+u_{2}^{2}-1$, respectively.

Choosing the first two terms in the series expansions of $u_{1}$ and $u_{2}$ as

41 $$\begin{aligned} u_{1,0}=1,\quad u_{1,1}=0,\quad u_{2,0}=0,\quad u_{2,1}=t. \end{aligned}$$

Then comparing the general terms on the left and right hand sides of (40), we derive the following recursion system

42 $$\begin{aligned} u_{1,n}&= L^{-1}\left( 2u_{2,n-2}-2A_{2,n-2}-B_{1,n-2}\right) , \\ u_{2,n}&= L^{-1}\left( 2u_{1,n-2}-2A_{1,n-2}-B_{2,n-2}\right) , \\ 0&= C_{n},\quad n\ge 2. \end{aligned}$$

Since $B_{k,n-2}=\sum _{i=0}^{n-2}u_{k,n-2-i}v_{i},\,k=1,2$ and $C_{n}=\sum _{i=0}^{n}\left( u_{1,n-i}u_{1,i}+u_{2,n-i}u_{2,i}\right)$, for $n\ge 2$, system (40) gives

43 $$\begin{aligned} u_{1,n}&= L^{-1}\left( 2u_{2,n-2}-2A_{2,n-2}-\sum _{i=0}^{n-2}u_{1,n-2-i}v_{i}\right) , \\ u_{2,n}&= L^{-1}\left( 2u_{1,n-2}-2A_{1,n-2}-\sum _{i=0}^{n-2}u_{2,n-2-i}v_{i}\right) , \\ 0&= \sum _{i=0}^{n}\left( u_{1,n-i}u_{1,i}+u_{2,n-i}u_{2,i}\right) ,\quad n\ge 2. \end{aligned}$$

System (43) can be written in the form

44 $$\begin{aligned} u_{1,n}+u_{1,0}L^{-1}v_{n-2}&= L^{-1}\left( 2u_{2,n-2}-2A_{2,n-2}-\sum _{i=0}^{n-3}u_{1,n-2-i}v_{i}\right) , \\ u_{2,n}+u_{2,0}L^{-1}v_{n-2}&= L^{-1}\left( 2u_{1,n-2}-2A_{1,n-2}-\sum _{i=0}^{n-3}u_{2,n-2-i}v_{i}\right) , \\ u_{1,0}u_{1,n}+u_{2,0}u_{2,n}&= -\left( 1/2\right) \sum _{i=1}^{n-1}\left( u_{1,n-i}u_{1,i}+u_{2,n-i}u_{2,i}\right) ,\quad n\ge 2. \end{aligned}$$

System (44) is a $$\left( 3\times 3\right)$$ linear algebraic system for the unknowns $$u_{1,n},$$$$u_{2,n}$$ and $$L^{-1}v_{n-2}$$, with the coefficient matrix

45 $$\begin{aligned} \left( \begin{array}{ccc} 1 &\quad 0 & \quad u_{1,0} \\ 0 & \quad 1 & \quad u_{2,0} \\ u_{1,0} &\quad u_{2,0} &\quad 0 \end{array} \right) . \end{aligned}$$

System (44) is uniquely solvable for all consistent initial conditions $u_{1,0}$ and $u_{2,0},$ since the determinant of this matrix is $-\left( u_{1,0}^{2}+u_{2,0}^{2}\right) =-1.$

Now, we solve system (44) recursively to obtain the solution of DAEs system (36)–(37).

For $n=2$, we have

46 $$\begin{aligned} u_{1,2}+L^{-1}v_{0}&= L^{-1}\left( 2u_{2,0}-2A_{2,0}\right) , \\ u_{2,2}&= L^{-1}\left( 2u_{1,0}-2A_{1,0}\right) , \\ u_{1,2}&= -\left( 1/2\right) (u_{1,1}^{2}+u_{2,1}^{2}). \end{aligned}$$

Since $A_{1,0}=u_{1,0}^{3}=1$ and $A_{2,0}=u_{2,0}^{3}=0$, system (46) reduces to

47 $$\begin{aligned} u_{1,2}+L^{-1}v_{0}&= 0, \\ u_{2,2}&= 0,\, \\ u_{1,2}&= -\frac{1}{2}t^{2}. \end{aligned}$$

Equations (47) yield

48 $$\begin{aligned} u_{1,2}=-\frac{1}{2}t^{2},\quad u_{2,2}=0,\quad v_{0}=1. \end{aligned}$$

For $n=3$, we have

49 $$\begin{aligned} u_{1,3}+L^{-1}v_{1}&= L^{-1}\left( 2u_{2,1}-2A_{2,1}-u_{1,1}v_{0}\right) , \\ u_{2,3}&= L^{-1}\left( 2u_{1,1}-2A_{1,1}-u_{2,1}v_{0}\right) , \\ u_{1,3}&= -(u_{1,2}u_{1,1}+u_{2,1}u_{2,2}). \end{aligned}$$

Since $A_{1,1}=3u_{1,0}^{2}u_{1,1}=0$ and $A_{2,1}=3u_{2,0}^{2}u_{2,1}=0$, system (49) reduces to

50 $$\begin{aligned} u_{1,3}+L^{-1}v_{1}&= \frac{1}{3}t^{3}, \\ u_{2,3}&= -\frac{1}{3!}t^{3}, \\ u_{1,3}&= 0. \end{aligned}$$

Equations (50) yield

51 $$\begin{aligned} u_{1,3}=0,\quad u_{2,3}=-\frac{1}{3!}t^{3},\quad v_{1}=2t. \end{aligned}$$

For $n=4$, we have

52 $$\begin{aligned} u_{1,4}+L^{-1}v_{2}&= L^{-1}\Big (2u_{2,2}-2A_{2,2}-\left( u_{1,2}v_{0}+u_{1,1}v_{1}\right) \Big ), \\ u_{2,4}&= L^{-1}\Big (2u_{1,2}-2A_{1,2}-\left( u_{2,2}v_{0}+u_{2,1}v_{1}\right) \Big ), \\ u_{1,4}&= -\left( 1/2\right) (2u_{1,3}u_{1,1}+2u_{2,3}u_{2,1}+u_{1,2}^{2}+u_{2,2}^{2}). \end{aligned}$$

Since $A_{1,2}=3u_{1,0}^{2}u_{1,2}+3u_{1,0}u_{1,1}^{2}=-(3/2)t^{2}$ and $A_{2,2}=3u_{2,0}^{2}u_{2,2}+3u_{2,0}u_{2,1}^{2}=0$, system (52) reduces to

53 $$\begin{aligned} u_{1,4}+L^{-1}v_{2}&= \frac{1}{4!}t^{4}, \\ u_{2,4}&= 0, \\ u_{1,4}&= \frac{1}{4!}t^{4}. \end{aligned}$$

Equations (53) yield

54 $$\begin{aligned} u_{1,4}=\frac{1}{4!}t^{4},\quad u_{2,4}=0,\quad v_{2}=0. \end{aligned}$$

For $n=5$, we have

55 $$\begin{aligned} u_{1,5}+L^{-1}v_{3}&= L^{-1}\left( 2u_{2,3}-2A_{2,3}-\left( u_{1,3}v_{0}+u_{1,2}v_{1}+u_{1,1}v_{2}\right) \right) , \\ u_{2,5}&= L^{-1}\left( 2u_{1,3}-2A_{1,3}-\left( u_{2,3}v_{0}+u_{2,2}v_{1}+u_{2,1}v_{2}\right) \right) , \\ u_{1,5}&=-(u_{1,4}u_{1,1}+u_{2,4}u_{2,1}+u_{1,3}u_{1,2}+u_{2,3}u_{2,2}). \end{aligned}$$

Since $A_{1,3}=3u_{1,0}^{2}u_{1,3}+6u_{1,0}u_{1,1}u_{1,2}+u_{1,1}^{3}=0$ and $A_{2,3}=3u_{2,0}^{2}u_{2,3}+6u_{2,0}u_{2,1}u_{2,2}+u_{2,1}^{3}=t^{3}$, system (55) reduces to

56 $$\begin{aligned} u_{1,5}+L^{-1}v_{3}&= -\frac{1}{15}t^{5}, \\ u_{2,5}&= \frac{1}{5!}t^{5}, \\ u_{1,5}&= 0. \end{aligned}$$

Equations (56) yield

57 $$\begin{aligned} u_{1,5}=0,\quad u_{2,5}=\frac{1}{5!}t^{5},\quad v_{3}=-\frac{4}{3} t^{3}. \end{aligned}$$

Continuing this process until $n=9$ and using the previously calculated expansion components (48), (51), (54), (57) we obtain the following ADM solution expansion components

58 $$\begin{aligned} u_{1,2}&= -\frac{1}{2}t^{2},\quad u_{1,3} =0,\quad u_{1,4} =\frac{1}{4!}t^{4},\quad u_{1,5} =0 \\ u_{1,6}&= -\frac{1}{6!}t^{6},\quad u_{1,7} =0,\quad u_{1,8} =\frac{1}{8!}t^{8},\quad u_{1,9} =0, \\ u_{2,2}&=0,\quad u_{2,3} =-\frac{1}{3!} t^{3},\quad u_{2,4} =0,\quad u_{2,5} =\frac{1}{5!}t^{5}, \\ u_{2,6}&=0 ,\quad u_{2,7} =-\frac{1}{7!}t^{7},\quad u_{2,8} =0,\quad u_{2,9} =\frac{1}{9!}t^{9}, \\ v_{0}&= 1,\quad v_{1}=2t,\quad v_{2}=0,\, \, v_{3}=-\frac{4}{3}t^{3},\quad v_{4}=0,\, \, \\ v_{5}&=\frac{4}{15}t^{5},\quad v_{6}=0,\quad v_{7}=-\frac{8}{315}t^{7}. \end{aligned}$$

Using values (41) and (58), we construct the approximate solution

59 $$\begin{aligned} u_{1}\left( t\right) ={{\sum _{n=0}^{9}}}u_{1,n},\quad u_{2}\left( t\right) ={{\sum _{n=0}^{9}}}u_{2,n},\quad v\left( t\right) ={{ \sum _{n=0}^{7}}}v_{n}. \end{aligned}$$

Expansions (59) are the first few terms of Taylor expansions, around $t=0$, of

60 $$\begin{aligned} u_{1}\left( t\right) =\cos t,\quad u_{2}\left( t\right) =\sin t,\quad v\left( t\right) =1+\sin 2t, \end{aligned}$$

which is the exact solution of DAE initial-value problem (36)–(37).

## Discussion

System of higher-index differential–algebraic equations (DAEs) still require new numerical and analytical methods to solve them efficiently. Such problems are known to be difficult to solve. In this paper, we developed a novel technique that applies the Adomian decomposition method (ADM) directly to solve a class of nonlinear higher-index Hessenberg DAEs. Our technique has successfully handled this class of DAEs without the need for complex transformations like index-reductions. The proposed method transforms these DAEs into easily solvable algebraic systems for the expansion components of the solution. To demonstrate the effectiveness of our technique, we solve one nonlinear index-three Hessenberg DAEs system with nonlinear algebraic constraints is solved. It is important to note that nonlinear algebraic constraints make the DAE more difficult to solve. In particular some transformations like those in Hosseini (2006b) cannot not be used. This example shows that the direct application of the ADM is a simple powerful technique to obtain the exact or approximate solutions of nonlinear higher-index Hessenberg DAEs. In the case we want to solve a DAE with an unknown solution, one way to measure the error for the approximate solution is to use the mean square residual (MSR) as in Benhammouda and Vazquez-Leal (2015) since the convergence of the method is still not shown.

## Conclusion

This work presents the analytical solution of a class of nonlinear higher-index Hessenberg DAEs using the Adomian decomposition method (ADM). A procedure for solving this class of DAEs is presented. For this class, the technique was tested on a nonlinear higher-index Hessenberg DAEs system with nonlinear algebraic constraints. The results obtained show that the method can be applied to nonlinear higher-index Hessenberg DAEs efficiently to obtain the exact or an approximate solution. On the one hand, it is important to note that these types of DAEs are difficult to solve and on the other, the direct application of the ADM was able to solve this class of nonlinear higher-index Hessenberg DAEs. Also, it is important to note that unlike previous works (Duan and Sun 2014; Çelika et al. 2006; Hosseini 2006a, b), our procedure does not make transformations to DAEs before applying the ADM to them. Our technique is based on a straightforward procedure that can be programmed in Maple or Mathematica to simulate real application problems. Finally, further work is needed to show the convergence of the proposed method, apply it with its modifications (for example a multistage ADM) to solve nonlinear higher-index Hessenberg partial differential–algebraic equations and other nonlinear higher-index DAEs.
